# Caring for someone with cancer in rural Australia

**DOI:** 10.1007/s00520-022-06857-2

**Published:** 2022-02-14

**Authors:** Kate M. Gunn, Melanie Weeks, Kristiaan J. J. Spronk, Chloe Fletcher, Carlene Wilson

**Affiliations:** 1 Department of Rural Health, Allied Health and Human Performance, University of South Australia, North Terrace, Adelaide, South Australia Australia; 2 Flinders Centre for Innovation in Cancer, College of Medicine and Public Health, Flinders University, Adelaide, South Australia Australia; 3 Cancer Council SA, Adelaide, South Australia Australia; 4 Freemasons Centre for Male Health and Wellbeing, The University of Adelaide, Adelaide, South Australia Australia; 5 School of Psychology, The University of Adelaide, Adelaide, South Australia Australia; 6 La Trobe University, Melbourne, Victoria Australia

**Keywords:** Cancer, Oncology, Carer, Caregiver, Rural, Remote, Regional

## Abstract

**Purpose:**

To explore the experiences of people caring for someone with cancer, while living in rural Australia, and the impact of the cancer-caring role on their well-being.

**Method:**

Eighteen adults in regional or remote (‘rural’) Australia who cared for a person with cancer took part in semi-structured telephone interviews. Participants were aged 32–77 years and mainly female (66%). Data were analysed using thematic analysis and an essentialist approach.

**Results:**

Eight themes were identified: (1) travel is hard, but supports are available; (2) frustration with systems that do not demonstrate understanding of the rural context; (3) the importance of lay and peer support; (4) the impact of access to trusted, local health care services; (5) the importance of access to rurally relevant information (particularly on relevant services and what to expect); (6) living with uncertainty and balancing loss with hope; (7) reluctance to seek or accept psychological support; and (8) the gendered nature of care.

**Conclusion:**

Rural cancer carers’ roles can be made easier by improving health systems and coordination to ease the burden of travel, providing information about available support and what to expect throughout cancer treatment that is relevant to the rural context, and increasing access to quality health, community, and support services, including palliative care, in rural areas. More training on the specific needs of rural patients and their carers is needed for urban health care professionals. Peer support groups may have particular value for cancer carers in rural settings, where there are known to be multiple barriers to accessing professional sources of psychosocial support.

The impacts of a cancer diagnosis ripple beyond the individual diagnosed [1]. Informal carers (an unpaid relative, friend, or spouse of the person with cancer [2]) often take on day-to-day care. Cancer is one of the most common health conditions in receipt of informal care in Australia [3]. Becoming an informal carer may happen unexpectedly, cost time [4] and money [5], and be socially and emotionally demanding [6]. Caring for someone with cancer is generally thought to be associated with greater caregiver burden than caring for the elderly or patients with other chronic health conditions, such as diabetes [7]. Day-to-day care may be labour-intensive (assisting with activities of daily living, providing transportation, preparing meals, etc.) and emotionally taxing (advocating for health care, providing emotional support, etc.) [8], and is often needed long after treatment ends [9]. Consequently, many carers experience poor quality of life and psychological distress, with some studies showing a greater negative emotional impact on carers than those diagnosed with cancer themselves [10, 11].

The caring role is further impacted by the geographical location of the carer and the person for whom they are caring. In Australia, about one-third of the population lives outside of metropolitan centres. In comparison with their urban counterparts, rural cancer patients face poor treatment outcomes [12, 13]; report poor physical health, low quality of life, and unmet supportive care needs [14, 15]; and experience a multitude of barriers to accessing local health services and psychosocial support [16–20]. Those who live more remotely may experience worse cancer outcomes still [21]. Studies indicate that distance to treatment is associated with poorer cancer outcomes [22, 23]. In Australia, due to low population density in rural areas and centralization of specialist medical services in metropolitan centres, people who live rurally often travel great distances to access cancer care [24]. This has repercussions for carers, whose role in rural and remote settings is likely to be more demanding than that of their urban counterparts, particularly as a result of isolation, poorer access to health care services, and the need for carers to accompany patients travelling to access specialized treatment and assist with coordination between metropolitan and rural healthcare systems [25]. Indeed, risk factors for caregiver burden include a higher number of hours spent performing the caring role, social isolation, and lack of choice in being a carer [26], all of which are likely to have a greater impact on those living rurally. In support of this, a 2016 study of Australian carers found that rural carers’ self-reported physical and mental health was significantly lower than age-matched population norms [27].

Despite the challenges thought to be faced by cancer carers in rural Australia, little research has been undertaken to understand their exact experiences and supportive care needs. Most data obtained has been derived from broader studies of patient experiences [e.g. 18, 28, 29, 30]. An exception is a recent study [25] investigating travel and decision-making surrounding cancer treatment from the perspective of rural cancer carers and urban-based social workers. While this study is informative, its scope is limited to travel-related issues; it is unclear whether or not data saturation was reached; and, due to sole recruitment from Victoria, an Australian state with comparatively high population density and good access to health services, findings may not be generalisable to carers living in less densely populated and serviced regions of Australia. Therefore, the present study builds on previous research through an in-depth exploration of the experiences of people caring for a person with cancer in regional and remote (‘rural’) Australia, and the impact of the cancer-caring role on their well-being, with a view to informing future initiatives to support this group.

## Method

### Study design and reflexivity

A qualitative phenomenological approach, underpinned by an essentialist epistemology, was used to explore the experiences of rural cancer carers. Semi-structured, one-on-one interviews were conducted via telephone. Interviews were conducted by MW, a female, mature-age honours student, herself the daughter of a cancer survivor. The topic guide (presented in Table 1) was informed by relevant literature and the clinical and lived experiences of the research team in navigating complex medical systems within the rural Australian context.

**Table 1:** Interview topic guide

1. Overall experience of being a carer/ tell me your story
2. Most difficult aspects of being a carer
3. Support provided to you while you were a carer
4. Greatest needs while being a carer
5. What you wish you knew earlier/ would tell a friend facing the same situation
6. How your experiences might have been different if living metropolitan environment
7. How your experiences may have been different if you were the opposite gender

### Participants

Participants were recruited via media release, flyers (general practitioner waiting rooms and cancer support groups), a local cancer support charity’s social media, personal contacts, and snowball sampling. Purposive sampling based upon level of remoteness (assessed using the Accessibility Remoteness Index of Australia) and the patients’ stage of cancer (stable, curative treatment, palliative treatment or deceased) was employed to ensure a wide range of carer perspectives. Eighteen carers (12 women, aged 32–77 years) were interviewed, including sixteen spouses, one adult daughter, and one mother of someone diagnosed with cancer. Four participants were working, 11 were previously retired or not working, and three had retired due to caring responsibilities. Participant demographics are described in Table 2.

**Table 2:** Demographic characteristics of rural cancer carers and their associated cared-for cancer patients (*N* = 18)

**Characteristics of participants**	*n*	%
Age (years), range (Min = 32, Max = 77), *M* = 66		
Gender		
Female	12	66.7
Male	6	33.3
**Characteristics of cared-for cancer patients**		
Time since diagnosis (Min = 4 months, Max = 12 years), *M* = 4.64 years		
Cancer type of cared-for patients		
Immune system	4	22.2
Bowel	4	22.2
Breast	2	11
Lung	1	5.5
Brain	1	5.5
Cervix	1	5.5
Prostate	1	5.5
Esophagus	1	5.5
Gall bladder	1	5.5
Unknown primary	1	5.5
Condition/cancer stage of cared-for patients at time of interview		
Stable condition	4	22.2
Active treatment with curative intent	3	16.7
Palliative treatment/care	4	22.2
Deceased	7	38.9
Accessibility and Remoteness Index of Australia (ARIA)		
ARIA 2/Inner Regional	1	5.6
ARIA 3/Outer Regional	10	55.6
ARIA 4/Remote	7	38.9

### Procedures and materials

Ethics approval was granted by the University of Adelaide, School of Psychology Human Research Ethics Subcommittee. The topic guide was pilot tested and revised prior to data collection. Participants were screened for rurality using the ARIA index prior to interview. The ARIA index defines five categories of remoteness based on their distance from ‘service centres’ (ARIA 1 = Major Cities, ARIA 2 = Inner Regional, ARIA 3 = Outer Regional, ARIA 4 = Remote, ARIA 5 = Very Remote [28]). Confidentiality was explained and informed written consent obtained prior to interview. Interviews (averaging 75 min) were audio-recorded and professionally transcribed verbatim. Data saturation was reached by the 18^th^ interview (no new themes were evident in the last three interviews), after which recruitment and interviewing ceased. Participants were given the opportunity to provide feedback on a summary of results (theme level). No amendments were suggested.

### Analysis

Data were analysed using thematic analysis, following Braun and Clarke’s [29] steps for coding and analysing qualitative data. Data were organized using NVivo 12 Plus software. Interview transcripts were checked against audio-recordings, then read and re-read for familiarity. Transcripts were coded using an inductive approach and descriptive themes were developed from the data rather than theory. Thematic meaning was derived from surface-level semantics, based on the essentialist assumption that the descriptions participants provided were direct insights into their experiences. This meant that data were interpreted with minimal inference, to present a rich description of participants’ experiences or perspectives in everyday language [30, 31]. Interview quotations were aligned to each theme to ensure that analysis and interpretation remained close to the original data. Themes were reviewed to ensure that data within them were coherent, and that there were clear and identifiable distinctions between themes. Data were coded by MW and reviewed by KG. Disagreements about codes or themes were discussed until consensus was reached. A detailed research diary was kept by the primary researcher (MW) throughout the research process, to help keep track of any assumptions or biases she held, and prompt discussions with other members of the team about how they may be influencing interpretations and coding of the data. Reporting was informed by the consolidated criteria for reporting qualitative research (COREQ) [32] and Braun and Clarke’s 15-point thematic analysis checklist [29].

## Results

Eight themes were identified, as detailed below.

1. Travel is hard, but supports are available

Rural cancer carers reported practical, social, and financial challenges while travelling long distances with their loved one to access specialist medical treatment. For example, they needed to ensure their car was in working order, they were able and confident to drive in the city, and arrangements were made so that their homes, properties, children, and animals were cared for in their absence.

Going away… leaving the Island and going away for six weekly treatment visits... because you had to organise things around the farm, you had to organise things with the car….. that was probably the most stressful thing. I mean country people are not as at home… I was struggling with peak hour traffic at times, and you didn’t know how long to get [to the hospital] and the traffic was horrible… visiting the mainland hospital was the most stressful part. (Participant 9, male, remote)

Financial challenges related to the need to stop work or pay others to maintain their business. Additional hidden expenses included having to buy meals and pay car parking fees while attending appointments in the city.

The extent to which these challenges were experienced varied with the carers’ personal resources (including their own health status), level of support at home, connections to people in the city, access to information, distance of travel, mobility of the person with cancer, familiarity with metropolitan driving, level of access to community transportation services, and their financial resources. Carers described support provided by various non-government cancer organisations as reducing the burden of travel. For example, subsidized supported accommodation (where other rural people going through similar experiences are co-located), transport between the accommodation and hospital, and access to useful information were all described as valued. Participants explained that services offered by community transport schemes also eased their travel burden (where available), particularly for the elderly and for those who are unable or unwilling to drive long-distances or in the city.

We stayed for the whole eight weeks, yes. So it was really really good. And it cost us absolutely nothing because the Cancer Council paid for half and PATS paid the other half. It takes a lot of weight off your shoulders and worry if you’ve got somewhere like that to go to… it’s really, really good. (Participant 13, male, outer regional).

Carers emphasized that they appreciated the provision of rurally based treatment options, specialist equipment, and tele-consultations with urban-based specialists. The inevitability of needing to travel for some medical purposes was, nonetheless, acknowledged.

2. Frustration with systems that do not demonstrate understanding of the rural context

While rural cancer carers were appreciative of the financial assistance provided through the Patient Assisted Transport Schemes (PATS), navigating the system (e.g. meeting requirements, justifying the need for an escort) was often described as both frustrating and distressing.

When people say to me ‘how are you going?’ or ‘how’s [first name]?’ and all of that sort of stuff and we talk about breast cancer… I say to them that the single most horrendous day I’ve ever had was a day I had at the PATS office. (Participant 6, male, remote)

Participants also expressed frustration with the limited extent to which urban-based medical specialists understood the constraints associated with rural residency. Instances of travel arrangements being compromised by specialists running very late, cancelling or changing appointments at short notice were described.

...a couple of times we’ve been up to Adelaide and they’ve been ringing to cancel [first name]’s appointment and I said ‘we’re nearly there’… see they didn’t think Mount Gambier was too far away. I said ‘It’s nearly five hours drive’… ‘Oh I didn’t know that’. (Participant 18, female, outer regional)

Conversely, carers reported greatly appreciating appointments being considerately scheduled to help them minimise travel and time away from home.

3. The importance of lay and peer support

Lay (family, friends, neighbours) and peer-to-peer (others with similar experiences) supports were highlighted as important in helping to manage the demands of caring for a person with cancer. The availability of lay support (often provided by friends and neighbors when adult children live at a distance) was described as a unique advantage of rural life.

Every time someone comes out to visit they will bring bread and milk or people come from town and they bring fruit and veg, so the country does have its positive for that, and pick up the kids from school or run them to childcare, all that. There’s a network there that you just don’t have in the city. (Participant 8, male, remote)

Conversely, two participants recounted being surprised and disappointed when community members distanced themselves, rather than offering the support they expected and needed. Carers also described support directed to the patient as valuable, particularly when it allowed them to take a break from their caring roles.

Cancer peer support groups were commonly described as a valuable source of emotional, social, practical (psychosocial) support, and information. They reportedly bring people going through similar experiences together, reducing the sense of isolation, which may be particularly valuable for those lacking more informal support networks.

It’s just a support group that if you need to talk to somebody you can ring anyone of each other… you know, if I was finding it hard with [first name] I could ring one of the others and have a talk to get it off your shoulders… It’s a relief group more than anything. (Participant 13, male, outer regional)

Local support groups were also viewed as a valuable way for carers to ‘give back’ by sharing their experiences and knowledge with new members. As well as being a source of support, it was highlighted that these groups are also a valuable source of information, which is highly valued due to some carers’ physical isolation to more traditional sources of information. However, it should be noted that many rural carers lacked awareness that these supports existed.

4. The impact of access to trusted, local health care services

Carers explained that their ability to access local, trusted, and responsive healthcare services, such as general practitioners (GPs) and palliative care providers, significantly impacted their caring role. Interactions with these services and implications of this care (including medical care, information provision, and reassurance) not only affected patients’ physical and mental health, but also carers’ ability to cope effectively.

If it weren’t for the palliative care showering [first name], they used to come out and help [first name] change and everything, help him, if I didn’t have that support behind me I think I would have been falling in a heap myself, if I didn’t have that support. (Participant 18, female, outer regional)

Carers explained that unsatisfactory experiences with rural healthcare services can cause distress, frustration, and feelings of isolation. Challenges reported include long waiting times to access highly regarded GPs, high turnover of rural GPs (inhibiting the ability to build trust), communication challenges, and sub-optimal referrals by GPs unfamiliar with the Australian healthcare system.

The availability and quality of palliative care services in rural areas were another source of concern. A lack of trained personnel and relevant information left carers feeling unsupported and distressed. However, when supportive and skilled palliative care (e.g. symptom management), information, and reassurance were forthcoming, distress was reportedly reduced, making the final stages of care more manageable.

...and the doctor told me that it’s only a matter of only three to four days once they resort to that, and it happened exactly as he explained. And of course she went. And I think I can honestly say that the staff in the [***] Hospital took probably 90% of the trauma out of the final days. (Participant 9, male, remote)

5. The importance of access to rurally relevant information

Access to relevant information throughout the cancer journey was also highly valued and reported to reduce distress. This included information on what to expect (medically, financially, psychologically, socially, practically, particularly in the palliative stages) and services and systems that were available to help.

We had no idea what we were in for. We had no idea of where we were going, and not being city people, we were petrified of going to the city, how were we going to get around? What are we going to do? And all that sort of stuff, it was just terrible. (Participant 3, female, remote)

She was very good… the first time that [first name] went to see her and I was with her, she straight away said.... ‘you need to get a disabled carpark... you need to apply for carer’s allowance... and you need to apply for a wheelchair’... she was straight on to it. (Participant 6, male, remote)

When information of this type was not provided, negative opinions of services and unnecessary distress resulted.

... It was just a terrible thing. I’ve never seen anyone die before... and I think this is something that I think carers in palliative care... they should be prepared for this is what they may have to expect... it was a tremendous shock and I don’t think I will ever forget...(crying).... (Participant 4, female, remote)

6. Living with uncertainty and balancing loss with hope

Another challenge reported by the rural carers was uncertainty about the future for them and the patient. It also related to uncertainty around health status, heightened while awaiting test results, medical opinions, and treatment options. This made planning difficult.

“Oh it’s just that you can’t plan a day out. You can’t plan a day. You can’t plan anything if you don’t know what’s going to happen. (Participant 15, female, outer regional)

Uncertainty contributed to a perceived need to balance feelings of loss and sadness associated with the cancer diagnosis, with a sense of hope for the future. Losses described by participants related to future life plans, including loss of retirement-related plans like travel. Seeing the illness change the person they cared for and the nature of their relationship, also led to a sense of sadness and loss.

The personality of the person you’re caring for changes and that’s only disease and drug related… they can’t help that. And so the person you might have lived with for the last 40 years, is different, as a victim of cancer, than they are when they’re well. (Participant 1, male, remote)

Participants articulated that further losses associated with the sense of personal identity, freedom, social activities, and— particularly in the case of men — work, can also be a consequence of the caring role. Where work was maintained, benefits were observed. The death of their loved one also had obvious emotional consequences.

Carers’ perceived need to ‘hold onto hope’, even where there is ‘nothing to feel hopeful about’, was also highlighted. Many participants spoke of having to remain optimistic for the sake of the patient and avoiding expressing anything that might put negative thoughts into the patient’s head.

Well, I’d probably put some negatives in her mind. I’ve got to be positive. I keep telling her ‘Another day to victory, another day to victory’, and I just don’t want to say to her ‘Oh mate, we’re not going to win this’. I would never show her any negativities at all. (Participant 12, male, outer regional)

7. Reluctance to seek or accept psychological support

Despite many rural carers acknowledging the negative impact their caregiving role had on their mental and emotional well-being, only two participants reported actively seeking psychological support, while one other reported using the Cancer Council Helpline and finding it very supportive and reassuring.

Carers reported refusing offers of professional emotional support, which they deemed unnecessary, time-consuming, and uncomfortable. Distress was viewed as transitory, and patients’ needs viewed as more important than their own.

No, someone did ring me from another local service and asked if I needed some sort of counselling. I can’t remember who they were… but I said no…. at the time, you know, I was just too busy really. And I just felt that it wasn’t about me really. (Participant 3, female, remote)

8. The gendered nature of care

Rural male and female carers described the types of support offered to them differently; for example, males described practical support they received (e.g. cooked meals, shopping, domestic help, respite), whereas females spoke more frequently of emotional and social support.

...she’s a very good friend to me and she was supportive… I could sort of let go with her… and sometimes we’d just be driving somewhere and I’d just burst into tears and… she would just listen, and you know, not want to solve it or say ‘I’ll do this or I’ll do that’… she’d just listen to me.” (Participant 2, female, inner regional)

Similarly, male and female carers described a tendency to focus on different caring tasks. Men spoke more about practical tasks (e.g. arranging travel, attending appointments, preparing meals, doing household chores), and women more about providing emotional support.

I guess I’m doing most of the housework, a lot of the housework and just those sorts of things I suppose and you know if she’s having a lie down for the afternoon, you know getting water to her or taking medication, little things like that. (Participant 8, male, remote)

Some male carers highlighted initial challenges associated with undertaking domestic tasks, notwithstanding self-reported success with performing them. Men without a focus outside of their caring role, such as those no longer working, described particular difficulty with the increased isolation associated with caring.

So when I dropped work, I dropped contact with 150 people. And I guess today I’m still struggling as to what the bloody hell to do with my time. (Participant 12, male, outer regional).

## Discussion

Several themes identified have been raised previously in broader research on the experiences of cancer carers. There is congruence with urban carers’ experiences with living with uncertainty [6, 33–35], experiencing ongoing personal loss [33, 36] and ‘anticipatory grief’ (i.e. grief experienced in anticipation of future loss) [33], and feeling as though they need to appear hopeful to support the patient [37], as well as the gendered nature of caring roles [38–42]. However, there are also unique challenges that rural carers face that are worth highlighting, and some of the issues they share with their urban counterparts, appear to be experienced slightly differently in the rural context. For example, male carers described performing practical tasks such as arranging travel, preparing meals, and doing household chores, whereas female carers described providing emotional support. Although this is observed in carers more generally [38–42], it is likely to be more pronounced in rural areas where traditional gender-based roles are known to remain dominant [43].

Rural carers in the present study also reported particular distress from uncertainty relating to medical care, such as waiting for information about diagnoses, medical updates, treatment decisions, and prognoses. For rural carers, the experience of uncertainty is further impacted by the challenges associated with having to travel long distances to respond to changing illness circumstances (e.g. last-minute changes to travel and accommodation plans to facilitate further testing or undergo urgent treatment).

The findings from the present study confirm the importance of supported accommodation facilities for rural people affected by cancer [44, 45] (including carers) and the need for greater recognition of travel-related challenges by metropolitan health services [17, 25]. Although rural cancer carers expressed appreciation for patient travel subsidy schemes, they also described difficulties and frustration in navigating complex systems to access these supports (described elsewhere for patients with cancer [25, 46, 47] and other serious health conditions [48]). Frustrations extended to processes and systems that carers perceived did not reflect understanding of their rural context, and likely compounded travel-related burden. Travel-related burden was also found to be influenced by the carers’ own health and social supports, their familiarity with metropolitan driving, mobility of the person with cancer, travel distances, and the accessibility of community transportation services and/or financial assistance.

Similarly, rural cancer carers indicated a lack information relevant to their rural context, particularly about what to expect when caring, supporting someone travelling for treatment, and support available in their locality. This is consistent with previous reports from rural cancer patients [17, 18, 49], but importantly highlights the implications of this gap for rural carers too. Improving awareness of, and access to, quality rural health care services, including palliative care services, will help ameliorate this issue, and could help reduce distress and burden. The provision of rurally focused cancer information on websites and in video format may help meet this need [50, 51].

Rural cancer carers also described the importance of having access to trusted health care services in local (rural) locations, and importantly highlighted the ways in which systems and models of care could be improved— to the benefit of both rural patients *and* carers, and via the use of lay and peer-to-peer supports. Peer support groups provide a sense of community, acceptance, and access to information and support unavailable from other sources [52]. Rural carers in this study indicated the value of peer supports in minimizing the risk of social isolation and providing an educational resource. Due to multiple barriers to accessing professional psychosocial support in many rural areas, they may play a particularly important role in these settings. Their value to rural cancer *patients* has been noted previously [18]. Other rural Australian populations have also indicated that this model would facilitate help-seeking from professional mental health practitioners [53]. Some carers in the present study were not aware of peer support groups; therefore, greater awareness of such groups and their availability in rural and remote regions could reduce caregiver burden.

A significant strength of this study is the use of purposive sampling to ensure that participants represented a range of regional and remote experiences and stages of the cancer trajectory. While the purpose of qualitative research is not to generate generalizable findings, it is noted that the findings may still have been impacted by self-selection bias, based upon other variables. For example, those who had more challenging experiences, more extreme views, more time to participate in research, or higher levels of education may have been particularly motivated to participate in this study (like all research). The intention of this work was to broadly explore the experiences of rural cancer carers and the impact of the cancer-caring role on their well-being, with a view to highlighting potential intervention targets that may inform future initiatives to support this group. These could be tested using quantitative designs and samples that have been carefully selected to ensure they are representative of the broader population based upon a larger number of variables, in the future.

In summary, cancer carers living in rural or remote Australia experience a range of challenges that are associated with their need to support patients to travel to attend medical appointments and access treatment, frustrations with systems that often fail to demonstrate understanding of their rural context, and difficulties accessing trusted, local health care services and rurally relevant information. These may, at least in part, be addressed through training for urban-based health care professionals on the specific needs of rural patients and their carers. Improvements to travel subsidy systems, metropolitan hospital appointment scheduling, information provision, and the quality and availability of health care services in rural areas would also be of great benefit to this group. Furthermore, lay and peer supports are valued by rural cancer carers and may be particularly valuable to help to fill gaps in rural settings where there are multiple barriers to accessing professional psychosocial support.

## Data Availability

We have control of all primary data and agree to allow the journal to review our data if required.
